# *Rhodotorula mucilaginosa* (A. Jörg.) F.C. Harrison 1928 and onychomycosis: three case reports for an unusual and underestimated combo

**DOI:** 10.1093/femsle/fnaf089

**Published:** 2025-08-18

**Authors:** Mirko Benvenuti, Martina Burlando, Emanuele Claudio Cozzani

**Affiliations:** Section of Dermatology, Department of Health Sciences (DISSAL), University of Genoa, 16132 Genoa, Italy; Section of Dermatology, Department of Health Sciences (DISSAL), University of Genoa, 16132 Genoa, Italy; Section of Dermatology, Department of Health Sciences (DISSAL), University of Genoa, 16132 Genoa, Italy

**Keywords:** Rhodotorula mucilaginosa, onychomycosis, Pseudomonas, yeast

## Abstract

Here we describe the case reports of three patients, 35, 64, and 72 year old, respectively, who were presented to the dermatology clinic for suspected onychomycosis with discolored and hardened nails. Both light microscopic analysis of the samples taken and culture analysis on SDA and DTM media were carried out. Direct microscopic analysis revealed the presence of yeast cells in the interstitial space of keratinocytes. Culture examinations resulted in the isolation of yeasts of the genus *Rhodotorula*. Subsequent metabolic and morphological analysis was conducted to ascertain the identification of the genus and species as *Rhodotorula mucilaginosa*. The involvement of this opportunistic pathogen in cases of onychomycosis is considered very rare and usually regarded as incidental; however, it should not be ruled out as a possible cause in favor of the more common dermatophytes or *Candida* spp. infections.

## Teaser

Rhodotorula is a genus of carotenoid producing yeasts, usually not associated with onychomycosis. Here we report three different cases of nail infection caused by this genus.

## Introduction

Fungal infections, or mycoses, fall under the classic classification of diseases caused by fungi in humans (Benvenuti et al. 2024). *Rhodotorula* is a genus of carotenoid-producing yeasts, belonging to *Basidiomycota*, characterized by small cells and, rarely, rudimentary pseudohyphae, largely distributed in the environment, and frequently isolated by different matrices such as air, soil, lakes, ocean water, different foods as well as often as part flora of the human gastrointestinal, respiratory and genital system (Wirth and Goldani 2012, Ioannou et al. 2019). This genus has a strong affinity for plastic materials, colonizing different surfaces, such as dialysis equipment, fiber-optic bronchoscopes, shower curtains, bathtubs, and toothbrushes (Kiehn et al. 1992, Hagan et al. 1995) and therefore causing opportunistic infections in hospitalized and generally immunocompromised individuals. Here we present case reports of three patients who were presented, in the span of 6 months, to the dermatology clinic for suspected onychomycosis. Culture examinations resulted in the isolation of yeasts of the genus Rhodotorula. Subsequent metabolic and morphological analysis was conducted to confirm the identification of the genus and species.

## Cases presentation

### Case 1

A 35-year-old female was evaluated at the dermatology clinic for persistent changes in the appearance of one of her fingernails. She reported a progressive discoloration and hardening of the nail over the past several months, without associated pain, pruritus, or discharge. The patient expressed concern about the aesthetic appearance and recent increased rigidity of the nail, which had become more noticeable during daily activities. On physical examination, the affected fingernail appeared thickened, hardened, and diffusely brownish in color, with no signs of periungual inflammation or subungual debris. The surrounding skin was intact and nonmacerated, and there were no signs of onycholysis or trauma. The nail surface was irregular, but the lunula remained partially visible. The patient denied any relevant past medical history, including diabetes, immunosuppression, or peripheral vascular disease. She also reported no known risk factors for onychomycosis, such as frequent gardening, use of communal facilities (e.g. gyms or swimming pools), habitual nail biting, or prolonged glove wear. She had not undergone recent manicures or artificial nail applications, and there was no history of nail trauma. Given the chronicity of symptoms, the nail's altered morphology, and the absence of external contamination or debris, a fungal etiology was considered.

### Case 2

**Figure 1.**
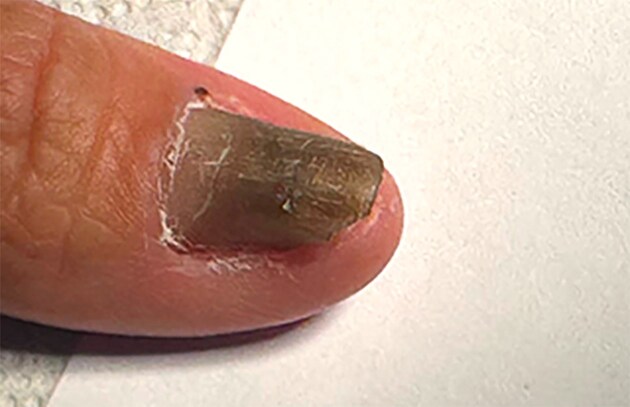
Infected nail of one of the patients, please take note of the staining reminiscent of other conditions such as green nail syndrome or infections with other nondermatophyte fungi such as *Fusarium* spp. or *Aspergillus* spp.

A 64-year-old male was referred to the dermatology clinic for evaluation of progressive nail changes Fig. 1. The patient reported a history of type 2 diabetes. Physical examination demonstrated dystrophic and hardened nails with the absence of onycholysis and dark greenish brown discoloration, reminiscent more of a green nail syndrome from *Pseudomonas aeruginosa* than a mycosis. However, upon detailed clinical evaluation, several key features typically associated with green nail syndrome were absent, including onycholysis, periungual maceration, and the characteristic softening of the nail plate. Instead, the nail appeared dystrophic and hardened, deviating from the classical presentation of a *Pseudomonas*-related infection. These differences in clinical presentation, particularly the absence of onycholysis and maceration, led us to consider onychomycosis as a more likely diagnosis

### Case 3

A 72-year-old female was referred to the dermatology clinic with complaints of progressive nail thickening, discoloration, and crumbling, predominantly affecting the toenails. The patient reported increasing discomfort while walking and difficulties with trimming her nails over the past several months. Her medical history was significant for advanced osteoarthritis, particularly involving the knees and hips, which had led to markedly reduced mobility and difficulty in maintaining regular foot care. The patient also lived alone and reported challenges with self-care, including inadequate nail hygiene and infrequent foot inspections. These factors, combined with the closed and moist environment typically associated with prolonged footwear use, placed her at an increased risk for onychomycosis. On physical examination, multiple toenails appeared thickened, opaque, yellowish-brown in color, brittle, and friable, with subungual debris and loss of normal nail architecture features strongly suggestive of fungal nail infection. No signs of acute inflammation or periungual cellulitis were observed.

## Materials and methods

For each patient, samples taken from the nails via blade removal were grown on dextrose Sabouraud SDA agar prepared in the laboratory in the following recipe: agar 10.0 g, peptone 10.0 g, D(+)glucose 5.0 g, chloramphenicol 0.1 g, and cycloheximide 0.5 g were added per 0.5 L of distilled water, Christensen's Urea Agar was prepared with the same recipe, adding urea 20 g and phenol red 0.012 g, and Dermatophytes Selective Agar (DTM) purchased from Merck Italy via Monte Rosa 93–20 149 Milano (MI). The inoculated plates were kept at a temperature of 25°C and high relative humidity, following 12-hour light-dark cycles until complete growth of the myocytes. Microscopic analyses of the grown cultures were conducted after 2 weeks through a direct scoop and staining with lactophenol blue. To have correct identification as Rhodotrula sp. successive analyses were conducted. Following this flow chart.

Sample culturing—incubation:

4–7 days—media:—SDA: growth observed (shiny, smooth, orange-pink, yeast-like)—DTM: no growth

Microscopic observation—stain:

Lactophenol blue—morphology:—ovoid, small yeast cells (average 3 × 8.5 nm)—apical budding—no true hyphae; rare pseudohyphae—no capsule

Further identification:

One colony per patient suspended in PBS and re-seeded on SDA plates—growth observations:—at 37°C: slowed growth, no carotenoids—at 25°C: resumed orange-pink pigment production—Christensen's urea agar:—pink color change after 12 hours—indicates strong urease activity

Conclusion identification confirmed:

*Rhodotorula mucilaginosa* based on morphological and metabolic data

## Results

**Figure 2.**
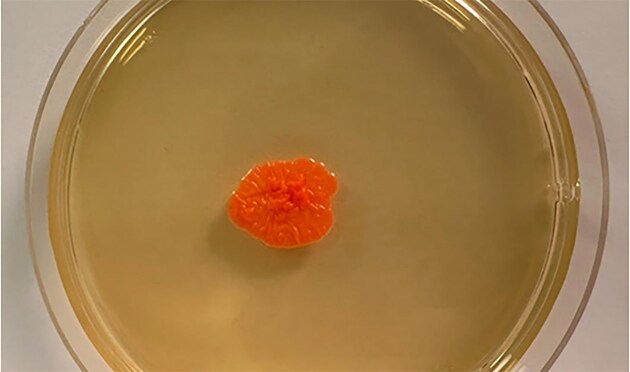
Appearance of *Rhodotorula mucilaginosa* colony isolated from one of the patients and grown on SDA.

**Figure 3.**
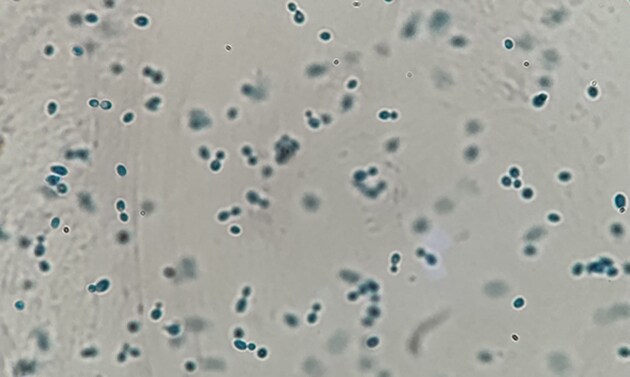
Emulsion of sampled and cultured *Rhodotorula mucilaginosa*, stained with cotton bleu, under a microscope. The budding point of the yeast cells is apical, and the shape is ovoid and rather elongated. No hyphae or pseudohyphae are present.

Cultures developed within 4–7 days, on SDA only, no growth on DTM, with a shiny, smooth, typically yeast-like aspect, with orange-pink color very well evident Fig. 2. In one of the cases, there was co-growth of a yeast of the genus *Candida*. Direct microscopic observation of the cells stained with lactophenol blue allowed determination of morology: the yeast cells appeared as ovoid, small, with an average size of 3 × 8.5 nm, characterized by apical budding, absence of hyphal from, and at most some occasional pseudohyphae. The presence of a capsule was not detected Fig. 3. For all three cases, combined antifungal therapy consisting of oral terbinafine 250 mg/die and topical ketoconazole for 4 weeks, as it's the most promising treatment for yeasts that are known to have sometimes innate resistance to conventional therapies (Campitelli et al. 2017). All patients showed complete remission at the end of the treatment.

## Discussion

Onychomycosis is one of the most common disorders afflicting patients of all ages (Zaara et al. 2024). Numerous factors make it difficult to correctly diagnose and identify the etiologic agent in cases of onychomycosis, other symptoms associated with this pathology are common to other conditions such as trauma dystrophies, psoriasis, *Pseudomonas* green nail syndrome, and nail growth defects (Allevato 2010, Ohn et al. 2020, Canal-Garcia et al. 2022, Dumontier and Braga da Silva 2024) or even more rare nail disorders (Schneider and Tosti 2015). Fungi that cause onychomycosis include numerous species belonging to different genera that are not closely related. Epidemiological studies indicate that the main causes are dermatophytes belonging to the genus *Trichophyton*, in particular *T. rubrum* and secondarily *T. mentagrophytes*, yeasts of the genus *Candida*, nondermatophytic molds such as *Scopulariopsis brevicaulis* and *Aspergillus* spp. (Moreno and Arenas 2010, Gupta et al. 2020). Based on the clinical morphology of the affected nails, *Pseudomonas aeruginosa* was excluded as the likely causative agent. Although some cases may exhibit greenish-brown discoloration reminiscent of Green Nail Syndrome, characteristic features such as onycholysis, periungual maceration, and nail softening—typically associated with *P. aeruginosa* infections—were notably absent. Instead, the presentation was more consistent with dystrophic and hardened nail plates suggestive of a fungal etiology. Nevertheless, we acknowledge that the absence of targeted culture on selective media for *P. aeruginosa* (e.g. cetrimide agar) represents a limitation of the study, as it prevents definitive microbiological exclusion of this pathogen.

## Conclusion

*Rhodotorula* is a genus of pathogen rarely isolated in cases of onychomycosis (Zhou et al. 2014) and is principally considered a pathogen related to cases of catheter or foreign body fungemia, albeit with high mortality rates (Arendrup et al. 2014). The involvement of this an other opportunistic pathogens in cases of onychomycosis is considered very rare and incidental (Idris et al. 2020, Benvenuti and Cozzani 2025); however, our three case reports identified in the last months make it clear that it should not be excluded as a possible cause in favor of the more common dermatophytes or *Candida* spp. infections.
